# Abdominal wall necrotizing fasciitis as a complication of strangulated hernia - an ominous consequence of a preventable scenario

**DOI:** 10.1093/jscr/rjad417

**Published:** 2023-07-17

**Authors:** Elias Edward Lahham, Maram Albandak, Mohammed Ayyad, Mohammad AlQadi

**Affiliations:** Faculty of Medicine, Al-Quds University, Jerusalem, Palestine; Faculty of Medicine, Al-Quds University, Jerusalem, Palestine; Faculty of Medicine, Al-Quds University, Jerusalem, Palestine; Department of General Surgery, Beit-Jala Hospital, Bethlehem, Palestine

**Keywords:** strangulated hernia, necrotizing fasciitis, incisional hernia

## Abstract

The incidence rate of abdominal wall necrotizing fasciitis (NF) is low; however, it carries a high mortality rate. It can arise as a complication of a strangulated hernia, where a part of the intestine becomes trapped and deprived of its blood supply. Rarely, this can result in abdominal wall fasciitis, which carries a grim prognosis. Timely debridement, however, has been shown to yield improved outcomes. Here, we report our experience with a 53-y-old morbidly obese patient who presented with colicky abdominal pain lasting for 1 week, progressively worsening and becoming constant. She also exhibited symptoms of fever, constipation, vomiting and anorexia. Furthermore, there was an infected wound measuring 20 cm × 13 cm along the midline of the abdomen. Imaging studies revealed indications of small bowel obstruction. This case describes a unique presentation of strangulated incisional hernia complicated by NF of the anterior abdominal wall, successfully managed with surgery.

## INTRODUCTION

Abdominal wall fasciitis is a rare complication of perforated abdominal wall hernias. Although perforation is uncommon, delayed detection and treatment of strangulated hernias drastically increase the risk of this complication. The perforation often results in the translocation of intestinal bacteria into adjacent soft tissues with subsequent invasion leading to sepsis, abscess, fistula formation or necrotizing soft tissue infection [1]. Initially, strangulated hernias and necrotizing fasciitis (NF) may exhibit mild skin changes and non-severe complaints, making it difficult to distinguish between the two solely on a clinical basis. The underlying tissue necrosis can extend far beyond what is grossly visible due to the rapid and aggressive propagation of the underlying infection explaining its high mortality rate. Although the concurrent occurrence of both conditions is uncommon, it carries poor prognostic outcomes due to frequent delays in diagnosis and management [2].

## CASE PRESENTATION

**Figure 1:**
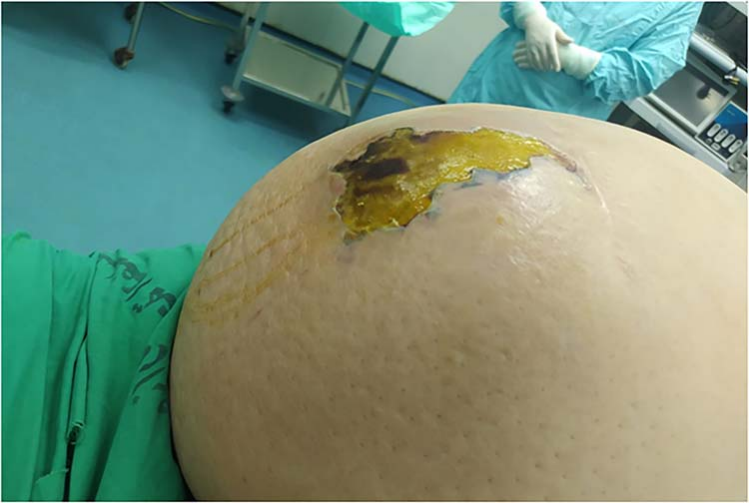
Necrotizing soft tissue infection of the anterior abdominal wall.

**Figure 2:**
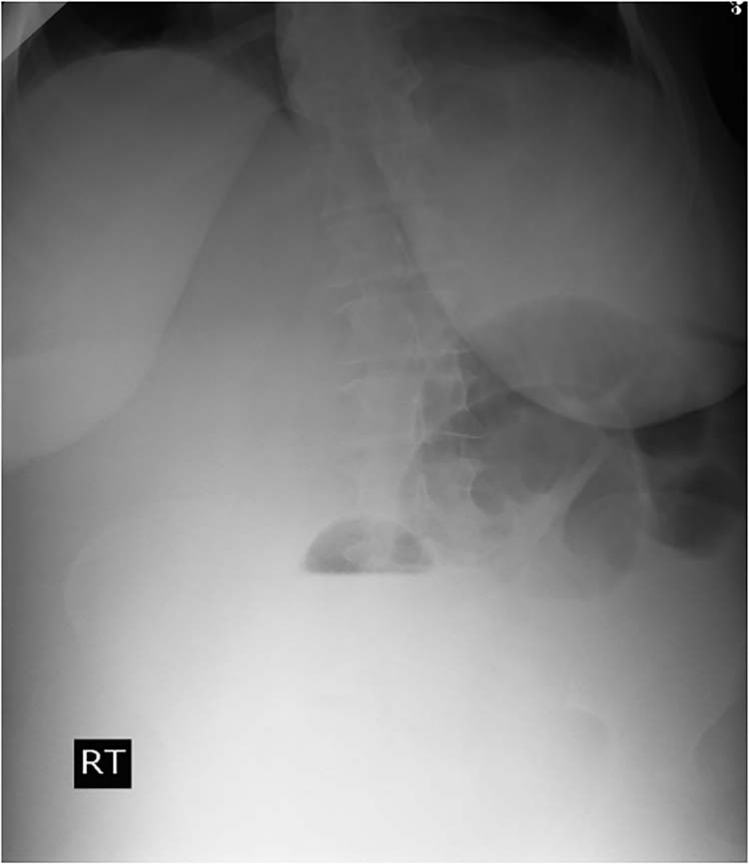
Plain abdominal X-ray demonstrated air-fluid levels of the small bowel.

**Figure 3:**
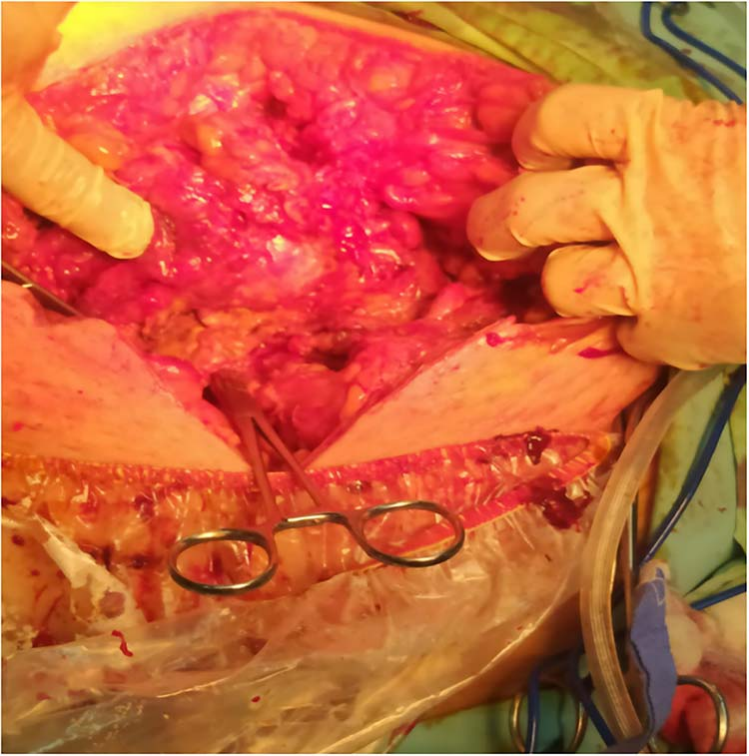
A left paramedian laparotomy incision.

**Figure 4:**
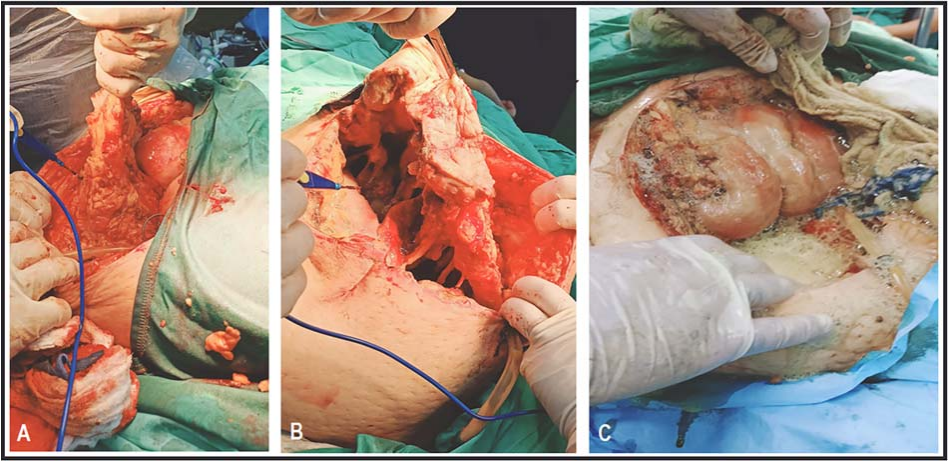
Surgical debridement and fasciotomy of the anterior abdominal wall.

**Figure 5:**
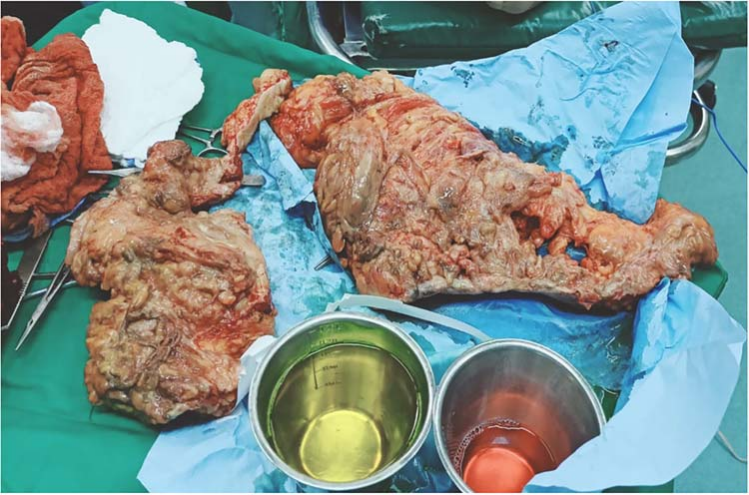
Gangrenous anterior abdominal wall resected during surgery.

**Figure 6:**
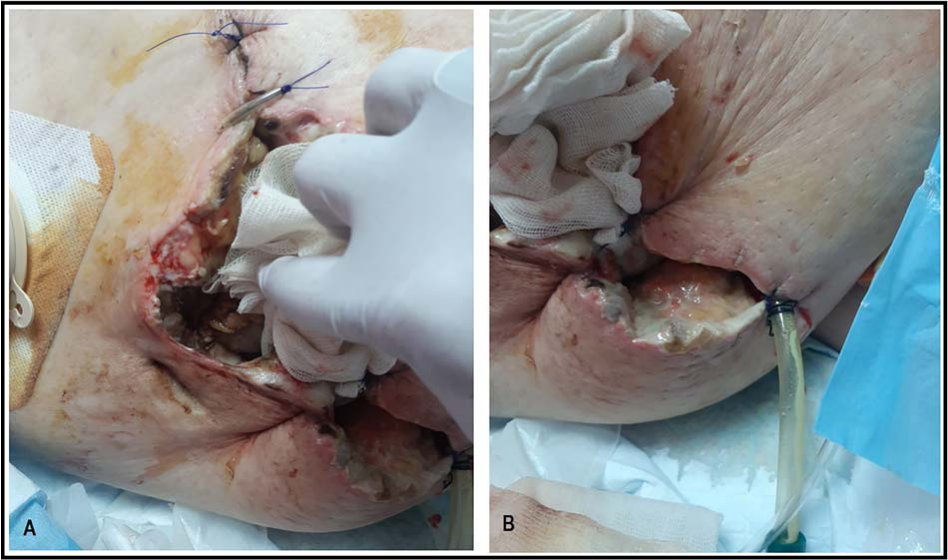
Suture approximation technique was done. At the site of maximum skin necrosis, the wound is left open for secondary healing, with a drain inserted in the abdominal wall.

**Figure 7:**
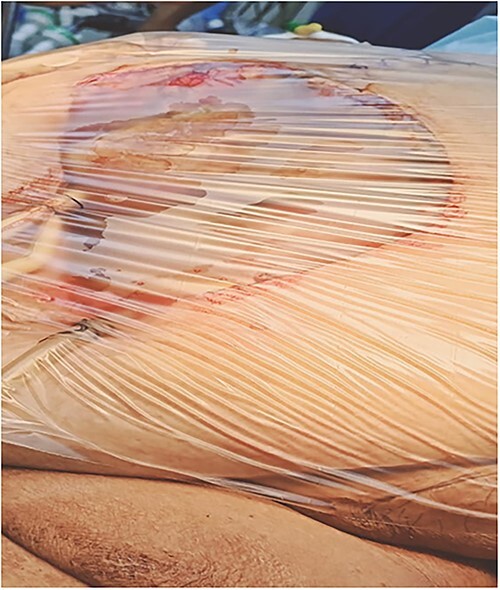
A plastic sheath over the exposed wound.

A 53-y-old morbidly obese female patient (BMI = 50) was diagnosed with colicky abdominal pain that progressively worsened over 1 week and became constant, without clear exacerbating or relieving factors. She also complained of fever, constipation, nausea, vomiting and anorexia. Past surgical history was significant for a surgically repaired umbilical hernia 10 y ago. The pain gradually became continuous and more severe, warranting hospital admission for further evaluation. On physical examination, the patient appeared unwell, dehydrated, and severely distressed. Vital signs showed a blood pressure of 100/60 mm Hg, pulse of 90 bpm and a temperature of 38 °C. Oxygen saturation was 95% on ambient air. Abdominal examination revealed midabdominal tenderness with mild abdominal distension, as well as high-pitched bowel sounds. Notably, there was an infected wound measuring 20 cm × 13 cm on the midline of the abdomen with signs of poor healing, tissue necrosis, and foul-smelling discharge (Fig. 1). Rectal examination was inconclusive. Laboratory tests indicated elevated levels of inflammatory markers, including a C-reactive protein (CRP) level of 445 mg/L and a white blood cell count of 30 000/ml. Hemoglobin level was 7.8 g/dl with a mean corpuscular volume (MCV) of 65. Additional abnormal results included a creatinine level of 1.8 mg/dl and a random blood sugar level of 310 mg/dL. Electrolyte levels were normal. Based on the laboratory findings, the LRINEC score was calculated to be 11, suggesting a high risk of NF. A plain abdominal X-ray showed fluid levels in the small intestine but no signs of pneumoperitoneum (Fig. 2). Abdominal ultrasound revealed localised swelling and fluid accumulation in the anterior abdominal wall, with dilated and edematous small intestine loops trapped within. The patient was ultimately diagnosed with strangulated incisional hernia complicated by NF of the abdomen. Management included nasogastric decompression, intravenous fluids and broad-spectrum antibiotics. Subsequently, the patient underwent emergency wound debridement under general anesthesia using a left paramedian laparotomy incision (Fig. 3). During the procedure, necrotic and dark skin, as well as friable subcutaneous tissue, were observed and resected (Figs 4  and 5). Approximately 500 ml of pus and digested food particles were drained from the abdominal wall. Controlled irrigation of the wound with saline was performed, and the strangulated bowel was identified, resected, and a loop ileostomy was created. There was no contamination of the peritoneal cavity. Two surgical drains were inserted, one in the abdominal wall and one in the peritoneal cavity. As the wound was considered contaminated, the fascia was sutured, but the skin edges were approximated and left open for secondary healing (Fig. 6). A temporary cover was applied using a plastic sheath (Fig. 7). The patient was then transferred to the intensive care unit (ICU) for continued postoperative wound care and debridement. On the 7th day, the patient was started on a soft diet and referred to the plastic surgery unit for ongoing wound treatment.

## DISCUSSION

The prevalence of incisional hernias is widespread, presenting a surgical challenge primarily due to their tendency to reoccur. Risk factors include smoking, obesity, and advancing age. Their occurrence is frequently attributed to inadequate wound healing or the failure of tissue to heal properly. Following abdominal surgery, incisional hernias represent one of the most prevalent complications, with a reported incidence as high as 10–20% following midline laparotomy [3]. NF is characterised by rapid microbial invasion of subcutaneous tissues or fascia, either due to external trauma or direct spread from a perforated viscus. Early symptoms include severe, disproportionate pain, with worsening symptoms, fever and sepsis occurring later in the disease course [4]. Early diagnosis of NF is challenging because of the non-specific clinical presentations, which often are the only early features. Importantly, early identification of NF, coupled with prompt and aggressive resuscitation and surgical debridement significantly improve outcomes [4, 5]. The diagnosis primarily relies on clinical assessment. However, when there is uncertainty, imaging, particularly computed tomography (CT), can provide valuable information, provided that does not hamper surgical intervention [6]. The Laboratory Risk Indicator for Necrotizing Fasciitis is a diagnostic tool used to assess the risk of NF. It incorporates several parameters, including white blood cell count, levels of hemoglobin, serum creatinine, sodium and glucose. The results should be interpreted in conjunction with clinical assessment and other imaging studies to ensure accurate diagnosis [5]. Due to the critical condition of our patient, a clinical diagnosis was established prior to proceeding to surgery. The mortality rates associated with necrotizing soft tissue infections reaches 25–46% [1]. Thus, high clinical suspicion is imperative for early intervention, as aggressive fluid resuscitation, prompt antibiotic administration and emergency surgical debridement are crucial for favorable outcomes in these cases.

## CONCLUSION

Early diagnosis and prompt management of strangulated hernias are vital in preventing the escalation of complications, such as abdominal wall fasciitis. Seeking immediate medical attention is imperative for individuals suspected of having a strangulated hernia to ensure timely intervention and avert further deterioration.

## Data Availability

The data used to support the findings of this study are included within the article.

## References

[1] Mwandri MB, Mwita JC, Bekele NA, Ali IM, Walsh MS. Anterior abdominal wall necrotizing fasciitis due to strangulated umbilical hernia: a diagnostic dilemma. Grand Rounds 2013;13:69–73.

[2] Akhtar M, Akhtar F, Bandyopadhyay D, Montgomery H, Mahomed A. Abdominal wall necrotizing fasciitis: a survivor from Meleney’s Minefield. Internet J Surg 2012;22:;1–6.

[3] de Oliveira Leite TF, Pires LAS, CAA C. Ventral incisional hernia case report: a therapeutic challenge. SAGE Open Med Case Rep 2020;8:2050313X2092762. 10.1177/2050313X20927624 PMC7268164 32537165

[4] Sarani B, Strong M, Pascual J, Schwab CW. Necrotizing fasciitis: current concepts and review of the literature. J Am Coll Surg 2009;208:279–88. 19228540 10.1016/j.jamcollsurg.2008.10.032

[5] Wong CH, Khin LW, Heng KS, Tan KC, Low CO. The LRINEC (Laboratory Risk Indicator for Necrotizing Fasciitis) score: a tool for distinguishing necrotizing fasciitis from other soft tissue infections. Crit Care Med 2004;32:1535–41. 15241098 10.1097/01.ccm.0000129486.35458.7d

[6] Zacharias N, Velmahos GC, Salama A, Alam HB, De Moya M, King DR, et al. Diagnosis of necrotizing soft tissue infections by computed tomography. Arch Surg 2010;145:452–5. 20479343 10.1001/archsurg.2010.50

